# Evaluation of Salivary Butylated Hydroxytoluene and Ascorbic Acid Levels in Type 2 Diabetes Mellitus Patients

**DOI:** 10.7759/cureus.56590

**Published:** 2024-03-20

**Authors:** Induja M, Roland Prethipa, Lokesh Kumar S, Rajalakshmanan Eswaramoorthy, Jayanth Kumar Vadivel

**Affiliations:** 1 Oral Medicine, Radiology, and Special Care Dentistry, Saveetha Dental College and Hospitals, Saveetha Institute of Medical and Technical Sciences (SIMATS), Chennai, IND; 2 Oral Medicine, Radiology, and Special Care Dentistry, Saveetha Dental College and Hospitals, Chennai, IND; 3 Biochemistry, Center of Molecular Medicine and Diagnostics, Saveetha Dental College and Hospitals, Saveetha Institute of Medical and Technical Sciences (SIMATS), Chennai, IND

**Keywords:** saliva, oxidative stress, diabetes mellitus, biomarkers, antioxidants

## Abstract

Background: Type 2 diabetes mellitus (T2DM) is a global health concern associated with systemic as well as oral complications. The preventive antioxidants found in saliva naturally reduce the damaging effects of reactive oxygen molecules. Any disruption to the regular functioning of these antioxidants may lead to oxidative stress, which could boost an individual's vulnerability to oral diseases. Diabetes patients are vulnerable to various dental complications, such as oral mucosal disorders, dental caries, dry mouth, and periodontal disease.

Aim: This study aimed to assess the salivary butylated hydroxytoluene (BHT) and ascorbic acid (AA) levels in patients with controlled and uncontrolled type 2 diabetes mellitus.

Materials and methods: The present study included samples from patients aged 45-65. Group I consisted of 20 controlled diabetic patients, and Group II consisted of 20 uncontrolled diabetic patients. Unstimulated whole saliva samples were collected from both groups, and laboratory analysis was done. Salivary BHT and AA levels were quantified using enzyme-linked immunosorbent assay (ELISA) and spectrophotometric assay.

Results: Salivary butylated hydroxytoluene levels were found to be higher in the uncontrolled diabetic group than in the controlled diabetic group, and salivary AA levels were found to be higher in the controlled diabetic group than in the uncontrolled diabetic group. The mean ± standard deviation (SD) values of butylated hydroxytoluene among controlled and uncontrolled diabetic patients were 2.98 ± 0.12 and 2.99 ± 0.11 absorbance units, respectively. The mean ± SD value of AA in the controlled group was found to be 2.99 ± 0.15 absorbance units, and the mean ± SD value of AA in the uncontrolled group was 2.64 ± 0.96 absorbance units. However, it has been found that there is no statistically significant difference between salivary BHT and AA levels among controlled and uncontrolled diabetics, with p-values of 0.867 and 0.419, respectively.

Conclusion: Values of salivary biochemical markers were distinctly different between controlled and uncontrolled diabetic groups. However, to establish a definite role of salivary BHT and AA levels as biomarkers in managing and monitoring type 2 diabetes, future studies are required, even though the trends exhibit possible alterations in biomarkers.

## Introduction

Diabetes mellitus (DM) is a condition that affects the body's metabolism and causes hyperglycemia due to a lack of insulin production or resistance to insulin. It contributes to significant implications for oral health, giving rise to various oral manifestations and complications such as periodontal disease, dental caries, poor wound healing, dry mouth, salivary dysfunction, candidiasis, and oral infections. These issues can harm a patient's quality of life, and chronic oral complications adversely affect blood glucose regulation [1]. Type 2 diabetes has a high global and national prevalence. Globally, 463 million cases of diabetes were projected in 2019 [2] with research predicting that the incidence of type 2 diabetes cases will rise by an additional 130-200 million cases by 2025-2030 [3]. In India, the highest diabetes prevalence was observed in Goa (26.4%), Puducherry (26.3%), and Kerala (25.5), and the prevalence of diabetes is 12.4% in Haryana [4].

Oxidative stress occurs when an unequal number of free radicals is produced compared to the body's ability to neutralize them. This phenomenon can lead to harmful effects on the body and has been implicated in the etiopathogenesis of various oral pathologies. Consequently, the body employs diverse mechanisms to produce antioxidants, endogenous and exogenous, which play a crucial role in averting diseases by counteracting the elevated levels of free radicals [5]. There is a clear correlation between dental problems and elevated HbA1c levels, which suggests that poorly controlled diabetes is a major risk factor for oral health [6]. Bashan et al. stated that DM, including type 1 and type 2 diabetes, causes an increase in free radical generation while decreasing antioxidant defense mechanisms. This imbalance eventually causes oxidative damage to biological components [7].

According to the National Institutes of Health, a biomarker is a measurable characteristic that indicates normal biological processes or a response to a therapeutic intervention, whether pathogenic or pharmacologic. It is essential to differentiate between screening biomarkers used to detect individuals at high risk for a disease, diagnostic biomarkers that confirm the presence, and predictive biomarkers that monitor the effectiveness of therapy [8]. Saliva was one bodily fluid used in the diagnosis process because the gold standard technique of monitoring the diabetes status involves blood investigations, which have an array of drawbacks [9]. The study aimed to assess the levels of salivary butylated hydroxytoluene (BHT) and ascorbic acid (AA) in patients with controlled and uncontrolled type 2 DM (T2DM). The objectives of the current study were to measure the unstimulated salivary levels of AA and BHT levels between controlled diabetics and uncontrolled diabetics and establish the correlation between them and the level of glycemic control.

## Materials and methods

Sample size calculation was done by keeping the power of the study at 90% and the significance level at 5% with an effect size of 0.5; the total sample size obtained was 40 [10]. Ethical clearance was obtained from the Institutional Human Ethical Committee (IHEC/SDC/OMED-2105/23/216) of Saveetha Dental College and Hospitals, Chennai, Tamil Nadu, India. This cross-sectional single-centered study recruited type 2 diabetic patients from the Institutional Special Care Dentistry unit. Seventy-five diabetic patients on treatment were recruited and screened based on the inclusion and exclusion criteria (Table 1). Forty participants were age and gender-matched and divided into two groups based on metabolic control as evidenced by plasma random blood sugar levels evaluated before the salivary sample collection. Patients with random blood sugar levels (7.8-11.0 mmol/L or ≥140-199 mg/dL) were categorized as group 1 (controlled type 2 diabetes group 20), and those with values ≥ 11.1 mmol/L or 200 mg/dL were classified as group 2 (uncontrolled type 2 diabetes group 20) [11].

**Table 1:** Inclusion and exclusion criteria of the samples in the current study

Inclusion criteria	Exclusion criteria
Patients diagnosed with type 2 diabetes mellitus and under medication	Patients with other systemic diseases coexisting with diabetes mellitus, a history of radiotherapy for head and neck cancer, and a history of salivary gland disorders
Patients falling under the age group of 45-65 years	Patients diagnosed with type 1 diabetes mellitus and gestational diabetes

Laboratory assessment

Sample Collection

Approximately 3 ml of unstimulated whole saliva was collected by allowing participants to drool naturally into a clean, sterile, calibrated universal bottle. The collected sample was centrifuged at the rate of 5000 rpm for five minutes. Then, the separated fluid part was transferred to fresh Eppendorf bottles and stored at a temperature of 20°C until analyzed.

Butylated Hydroxytoluene Analysis

In the collected salivary samples, BHT levels were assessed by the indirect method of using a hydrogen peroxide reagent. The concentration of hydrogen peroxide (H_2_O_2_) was quantified using the method described by Wolff in 1994 [12], which is centered around the peroxide-mediated oxidation of ferrous ions (Fe^3+^). This oxidation is followed by the reaction of Fe^2+^ with xylenol orange, forming a complex known as Fe^3+ ^xylenol orange.

The experiments were performed at room temperature (36.67°C). Typically, in a 96-well, flat-bottomed polystyrene microtiter plate, a 100-µl H_2_O_2_ standard solution was serially diluted. As the standard blank, the diluting medium H_2_O_2_ was utilized. After loading the plate with salivary samples (50 µl), butylated hydroxytoluene (50 µl) was added to each well, followed by the Wolff technique (1994) [13]. The absorption intensity was measured at wavelengths of 492 and 630 nm. The salivary levels of BHT were determined by comparing the absorbance in the samples to that of standard solutions of H_2_O_2_.

Ascorbic Acid Analysis

To measure the amount of AA in the salivary samples, the following reagents were used: 1.0 M sulfuric acid, 0.05 M iodine, and starch solution. First, 0.1 grams of the sample were accurately weighed and dissolved in a mixture of 100 ml of fresh water and 25 ml of 1.0 M sulfuric acid. Immediately, the solution was titrated with 0.5 M iodine solution using the starch solution as an indicator during the titration. Titration continued until a persistent blue-violet color was obtained, indicating the reaction's endpoint. To determine the amount of AA in the sample, divide the milliliter of 0.05 M iodine solution used in the titration by 0. AA (50 l) was quickly added to each well, followed by 0.008806 grams (8.806 mg) of AA [14].

% of Ascorbic Acid = (Burette Reading [BR] x Actual Molarity x 0.008806 x 100/0.05 x Weight of the Sample)

Experiments were performed at room temperature (36.67°C). A solution was diluted in a 96-well, flat-bottomed polystyrene microtiter plate. After loading the plate with salivary samples (50 µl), AA (50 µl) was added to each well. The absorption intensity was measured at wavelengths of 492 and 630 nm. The absorbance values denoted the AA levels left in the salivary samples.

The absorption intensity was measured using a Microplate ELISA Plate Analyzer, which is automatic, with a linear measurement range from 0.000 to 3.500 absorbance units (A) having four optional wavelengths of 405, 450, 492, and 630 nm.

Interpretation

**Figure 1:**
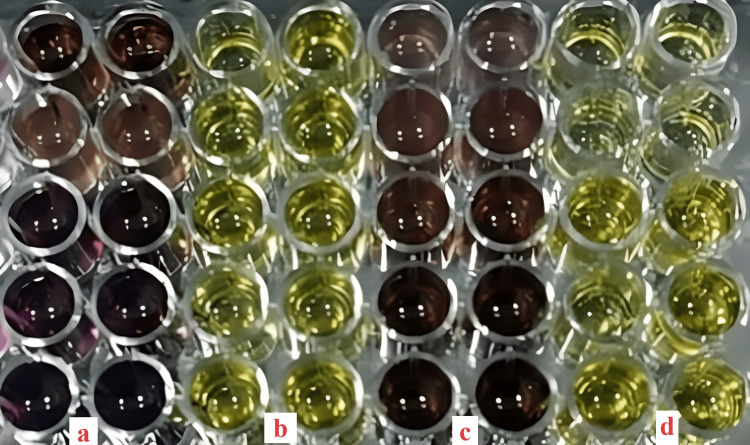
Laboratory analysis of saliva samples for ascorbic acid and butylated hydroxytoluene levels. Yellow denotes the salivary ascorbic acid samples, whereas brown denotes the butylated hydroxytoluene samples. Labels a and b indicate the samples of controlled diabetic patients subjected to butylated hydroxytoluene and ascorbic acid analysis, whereas labels c and d indicate the samples of uncontrolled diabetic patients subjected to butylated hydroxytoluene and ascorbic acid analysis.

The colorimetric assay is based on the fusion of the reagent with the compounds assessed in the salivary sample. The darker the color of the sample, the higher the concentration of BHT and AA in the evaluated samples, while the lighter the color, the lesser the amount of estimated salivary markers (Figure 1). Then, the ELISA plate was fed into the spectrophotometer (Robonik, ELISA Plate Analyzer) and assessed at wavelengths of 492 and 630 nm, and then the absorbance values were evaluated. The decrease in absorbance rate is denoted by lower values or vice versa.

Statistical analysis

Parametric statistical tests were utilized to assess the results. From the acquired data, an unpaired t-test was performed using SPSS v23.0 (IBM Corp., Armonk, NY), a statistical software for social sciences. The obtained data was presented in a summarized format as mean ± standard deviation (SD) of the mean. To compare the means between group 1 and group 2, an independent t-test was conducted. Statistical significance was determined by setting the threshold at p ≤ 0.05, indicating that results with significance were attributed to p-values that were equal to or less than 0.05. Pearson’s correlation test was performed to assess the relationship between BHT, AA, and random blood sugar values.

## Results

The study participants (n = 40) were gender-matched and divided into controlled and uncontrolled diabetic groups. The mean age of controlled and uncontrolled diabetic patients in the current study was 55.5 ± 1.20 years and 57.5 ± 1.35 years, respectively (Table 2).

**Table 2:** Gender distribution along with mean and standard deviation of the age of the participants

Study population	Total number of patients (n = 40)	Gender		Age, Mean ± SD (years)
		Male	Female	
Controlled diabetic group	20	10	10	55.5 ± 1.20 years
Uncontrolled diabetic group	20	10	10	57.5 ± 1.35 years

**Figure 2:**
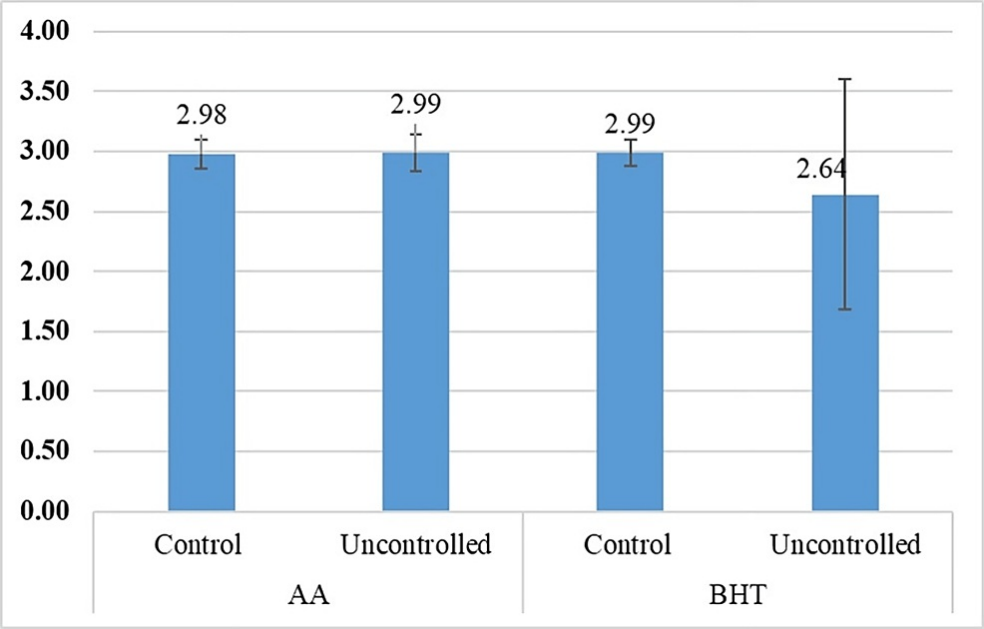
Distribution of mean ± standard deviation values of butylated hydroxytoluene (BHT) and ascorbic acid (AA) among the study population This figure depicts the mean ± SD values of BHT and AA among controlled and uncontrolled diabetic patients. The X-axis indicates the study groups, and the Y-axis indicates the mean absorbance values of AA and BHT. There is no statistically significant difference in BHT values in controlled and uncontrolled diabetic groups with p-value = 0.867. When comparing the mean ± SD values of AA among controlled and uncontrolled diabetic patients, no statistically significant difference (p-value = 0.419) was found.

The mean ± SD values of BHT among controlled and uncontrolled diabetic patients are 2.98 ± 0.12 and 2.99 ± 0.11 absorbance units, respectively. The mean ± SD value of AA in the controlled group was 2.99 ± 0.15 absorbance units. The mean ± SD value of AA in the uncontrolled diabetic group was 2.64 ± 0.96 absorbance units (Figure 2).

Tables 3, 4 show the correlation between BHT, AA, and random blood sugar (RBS) values in the controlled and uncontrolled diabetic groups. When Pearson’s correlation test was performed, a positive correlation was found between the BHT and RBS values in the controlled diabetic group, and this relationship was statistically significant (p-value = 0.01). No statistically significant correlation was found between the AA and RBS values in the controlled and uncontrolled groups.

**Table 3:** Correlation between the butylated hydroxytoluene (BHT) and glycemic values among the controlled and uncontrolled diabetic patients **Correlation is significant at p < 0.01. *Correlation is significant at p < 0.05.

Random blood sugar in controlled diabetic patients	Butylated hydroxytoluene in controlled diabetic patients	Random blood sugar in uncontrolled diabetic patients	Butylated hydroxytoluene in uncontrolled diabetic patients
1	0.910^*^	0.894^*^	-0.333
0.910^*^	1	0.950^**^	-0.153
0.894^*^	0.950^**^	1	0.048
-0.333	-0.153	0.048	1

**Table 4:** Correlation between the ascorbic acid (AA) levels and glycemic values among the controlled and uncontrolled diabetic patients No statistically significant correlation was found between salivary ascorbic acid and random blood sugar values in the controlled and uncontrolled diabetic groups.

Random blood sugar in controlled diabetic patients	Ascorbic acid in controlled diabetic patients	Random blood sugar in uncontrolled diabetic patients	Ascorbic acid in uncontrolled diabetic patients
1	0.692	-0.030	0.596
0.692	1	-0.345	0.215
-0.030	-0.345	1	0.675
0.596	0.215	0.675	1

## Discussion

Oxidative stress is a mediator of insulin resistance, leading to the development of glucose intolerance and the establishment of DM, subsequently favoring cell injury leading to diabetic complications. Beta cells are more vulnerable to reactive oxygen species (ROS) because they have lower concentrations of antioxidant enzymes. Thus, oxidative stress can disrupt mitochondrial function and drastically lower insulin production [15]. All substances that prevent or delay substrate oxidation, even at deficient concentrations, can be considered antioxidants. The body uses various mechanisms to produce antioxidants, either endogenous or exogenous. It helps to prevent diseases by shielding cells from their toxic effects and neutralizing the elevated levels of free radicals [16]. Al-Rawi concluded that measuring salivary oxidative stress markers has provided valuable insights into the causes and development of diabetes and suggested that antioxidants such as vitamins E and C could potentially assist individuals in enhancing the body's antioxidant defenses [10].

Antioxidants play a vital role in neutralizing harmful free radicals in the body, and vitamin C, being water-soluble, can work both outside and inside the cells. Low levels of vitamin C in the blood may contribute to oxidative damage and the development of disease conditions [17]. A 20-year follow-up analysis of the Dutch and Finnish cohorts of the Seven Countries Study showed an inverse relationship between vitamin C and glucose intolerance and suggested that antioxidants may play a role in the development of abnormalities in glucose metabolism [18]. Variations in vitamin concentrations can be helpful markers for evaluating diabetic disorders since vitamins like C, E, and A are essential components of antioxidant systems that help eliminate damaging oxidizing agents [19].

In the present study, the salivary AA levels in the controlled diabetic group were found to be higher than (mean ± SD value = 2.99 ± 0.15) those in the uncontrolled group, which had an AA mean ± SD value of 2.64 ± 0.96. Wilson et al. found individuals with type 2 DM had plasma vitamin C concentrations that were considerably lower than those with standard glucose tolerance (41.2 µmol/L versus 57.4 µmol/L, p < 0.05). Additionally, both the prediabetes and type 2 DM groups had a more significant proportion of vitamin C deficiency (i.e., <11.0 µmol/L) [20]. Previous literature proposed a few reasons for reduced plasma vitamin C levels in diabetic patients compared to those with standard glucose control: (1) Due to its molecular similarities to the oxidized form of vitamin C (dehydroascorbic acid), blood glucose may compete with vitamin C for absorption into cells; (2) increased oxidative stress has the potential to decrease antioxidant stores; (3) higher excretion of ascorbate may occur in those with microalbuminuria [18]. Bansal and Hadimani stated that people with diabetes have a higher need for vitamin C due to the preventive effects of dietary consumption of antioxidative vitamin C in avoiding oxidative damage. Vitamin C absorption in cells is hindered by hyperglycemia [21].

BHT administration in animals has demonstrated its ability to mitigate cataract formation induced by diabetes [22]. One explanation for how BHT postpones the development of cataracts brought on by diabetes may be related to protecting the lens cell membrane from oxidative damage, as described in the elaborate procedure proposed by Linklater et al. [23]. Creighton et al. [24] found that protective and chain-breaking antioxidant efficacy dramatically decreased in non-obese diabetic mice. Still, when BHT was added to the diet, the phenomena returned to normal. In the present study, the salivary BHT levels among controlled (mean ± SD value = 2.98 ± 0.12) were less than the uncontrolled diabetic patients (mean ± SD value = 2.99 ± 0.11).

Limitations and recommendations

The study had limitations, including limited sample size, single-centered design, and the use of RBS levels instead of the oral glucose tolerance test (OGTT) or glycated hemoglobin assay (HbA1C), which are more precise markers for assessing diabetes control. More extensive research with a multicentered analysis considering a larger sample size, using HbA1c/OGTT as a preferable choice of screening, is recommended. Future studies should look at the relationship between BHT and AA levels in saliva and serum in people with DM to establish saliva as an alternative helpful marker to serum for tracking the disease.

## Conclusions

The decrease in salivary AA and BHT levels indirectly reflects the ongoing oxidative stress leading to damage in type 2 diabetic patients. It helps to assess the disease activity and severity. Thus, the present study adds to the increasing corpus of literature about the possible use of salivary oxidative and antioxidant biomarkers in type 2 DM patients for early detection, tracking the course of the disease, stratifying risk, assessing the potential complications, and furthering research and development in diabetes management.
